# Serum Gelatinases Levels in Multiple Sclerosis Patients during 21 Months of Natalizumab Therapy

**DOI:** 10.1155/2016/8434209

**Published:** 2016-06-02

**Authors:** Massimiliano Castellazzi, Tiziana Bellini, Alessandro Trentini, Serena Delbue, Francesca Elia, Matteo Gastaldi, Diego Franciotta, Roberto Bergamaschi, Maria Cristina Manfrinato, Carlo Alberto Volta, Enrico Granieri, Enrico Fainardi

**Affiliations:** ^1^Department of Biomedical and Specialist Surgical Sciences, University of Ferrara, 44124 Ferrara, Italy; ^2^Department of Biomedical, Surgical and Dental Sciences, University of Milano, 20133 Milano, Italy; ^3^Department of General Neurology, National Neurological Institute C. Mondino, 27100 Pavia, Italy; ^4^Department of Morphology, Experimental Medicine and Surgery, University of Ferrara, 44124 Ferrara, Italy; ^5^Department of Neurosciences and Rehabilitation, S. Anna Hospital, 44124 Ferrara, Italy

## Abstract

*Background*. Natalizumab is a highly effective treatment approved for multiple sclerosis (MS). The opening of the blood-brain barrier mediated by matrix metalloproteinases (MMPs) is considered a crucial step in MS pathogenesis. Our goal was to verify the utility of serum levels of active MMP-2 and MMP-9 as biomarkers in twenty MS patients treated with Natalizumab.* Methods*. Serum levels of active MMP-2 and MMP-9 and of specific tissue inhibitors TIMP-1 and TIMP-2 were determined before treatment and for 21 months of therapy.* Results*. Serum levels of active MMP-2 and MMP-9 and of TIMP-1 and TIMP-2 did not differ during the treatment. The ratio between MMP-9 and MMP-2 was increased at the 15th month compared with the 3rd, 6th, and 9th months, greater at the 18th month than at the 3rd and 6th months, and higher at the 21st than at the 3rd and 6th months.* Discussion*. Our data indicate that an imbalance between active MMP-9 and active MMP-2 can occur in MS patients after 15 months of Natalizumab therapy; however, they do not support the use of serum active MMP-2 and active MMP-9 and TIMP-1 and TIMP-2 levels as biomarkers for monitoring therapeutic response to Natalizumab.

## 1. Introduction

Multiple sclerosis (MS) is a chronic inflammatory disease of the central nervous system (CNS) of presumed autoimmune origin that is characterized by demyelination and axonal loss [1]. MS affects young adults, women more frequently than men, and is clinically marked by exacerbations, called relapses, which typically show dissemination in space and time [2]. Brain inflammation is initiated and sustained by lymphocyte migration across the blood-brain barrier (BBB) [3]. In particular, the interaction of *α*4*β*1 integrin on the surface of lymphocytes with vascular-cell adhesion molecule 1 (VCAM-1) and on the surface of vascular endothelial cells in brain and spinal cord blood vessels mediates the adhesion and migration of lymphocytes in inflamed CNS sites [4]. Natalizumab (Tysabri, Biogen Idec Inc., Cambridge, Massachusetts, USA) is a humanized anti-*α*4 integrin monoclonal antibody approved for relapsing-remitting multiple sclerosis (RRMS) [5, 6]. The efficacy of Natalizumab monotherapy was demonstrated in clinical trials by the reduction in relapse rate and the progression of disability [7]. Consequently, Natalizumab is used as a second-line treatment in MS patients who have a suboptimal response to first-line disease-modifying therapies or as a first-line therapy in those with a highly active disease [8]. Notwithstanding the unquestionable benefits, anti-*α*4 integrin treatment is, however, associated with John Cunningham Virus- (JCV-) mediated progressive multifocal leukoencephalopathy (PML), a severe adverse event [9]. Although MS etiology remains largely unknown, the migration of immunocompetent cells into the CNS is dependent on several factors, but it fundamentally requires the opening of the BBB, a mechanism in which matrix metalloproteinases (MMPs) play a crucial role [10, 11]. These enzymes are a family of zinc-containing and calcium-requiring endopeptidases, which are secreted into extracellular space as a latent inactive proform that becomes activated through a proteolytic cleavage [12]. Specific tissue inhibitors of metalloproteinases (TIMPs) are molecules capable of binding either activated MMPs or their preforms and then finally of regulating MMP activity [13]. Due to their ability to degrade type IV collagen and gelatin, which are the main constituents of basal lamina, MMP-2 (gelatinase A, 72 kDa type IV collagenase) and MMP-9 (gelatinase B, 92 kDa type IV collagenase) are the most extensively studied subfamily of MMPs in the course of MS. In particular, previous studies demonstrated that cerebrospinal fluid (CSF) and serum levels of MMP-9 were higher in RRMS compared to progressive forms [14–16] and that elevated CSF and serum concentrations of MMP-9 were associated with clinical and magnetic resonance imaging (MRI) evidence of disease activity [15, 17–19] and disease evolution [20]. Moreover, CSF levels of MMP-9 were reduced after 12 months of Natalizumab treatment in 7 MS patients and in the same study CSF MMP-9 mean levels were higher in MS patients before Natalizumab treatment than in patients with other neurological diseases [21]. On the other hand, the significance of MMP-2 is more controversial in MS. In fact, while MMP-9 is predominantly upregulated in inflammatory conditions, MMP-2 is constitutively expressed in the brain [22]. Contradictory results have been reported in previous studies where MMP-2 levels in acute and chronic demyelinated lesions [23–25] as well as in CSF [26] in serum [15, 17, 20] and in peripheral blood mononuclear cells (PBMCs) [27–29] were increased, decreased, or represented in equivalent amounts in MS patients and in controls. In two previous studies we investigated the role of the active forms of MMP-9 and MMP-2 in MS and our results showed a reciprocal variation in these enzymes compared to the activity of the disease. In particular, serum and CSF levels and the intrathecal synthesis of active MMP-9 forms were associated with clinical and MRI disease activity [30] whereas CSF levels and intrathecal synthesis of active MMP-2 were more elevated in MS patients without MRI evidence of disease activity [31].

Considering these findings, in this study we, aimed to investigate serum temporal concentrations of active MMP-2 and active MMP-9 in a cohort of RRMS patients during 21 months of Natalizumab therapy.

## 2. Materials and Methods

### 2.1. Study Design and Sample Handling

The study design and the sample population were the same as in an earlier study [32]. Briefly, twenty consecutive RRMS [33] patients (17 female and 3 male) in treatment with Natalizumab were included in the study. All the patients were enrolled in the “Fondazione Istituto Neurologico C. Mondino” in Pavia. Serum samples were collected before starting therapy and then every three months for 21 months of treatment. All the samples were withdrawn, stored, and analyzed under the same conditions. At any time point: (a) disease severity was scored using Kurtzke's Expanded Disability Status Scale (EDSS) [34]; (b) presence of relapse was recorded as clinical activity; and (c) anti-JCV antibodies were determined to assess the risk of PML [35]. During the treatment, disability progression was defined as an increase of one point on EDSS score from baseline [5]. Brain MRI scans were performed at entry and at the end of the study and the occurrence of a new lesion on T2-weighted scans and/or a new gadolinium- (Gd-) enhancing lesion on T1-weighted scans was defined as MRI activity [33]. The approval of the Committee for Medical Ethics in Research was obtained for experiments involving human subjects. Written informed consent was obtained from all subjects participating in the study.

### 2.2. MMP-2 and MMP-9 Activity Assays

Serum levels of active MMP-2 and active MMP-9 were determined using commercially available specific activity assay systems as published before [30, 31] (Activity Assay System, Biotrak, Amersham Biosciences, Little Chalfont, UK; code RPN2631 and code RPN2634, resp.). With these methods, only circulating active forms of MMP-2 and MMP-9 were measured. All the reagents and standards were included in the kits. Briefly, in both activity assays, serum samples were applied in duplicate into 96-microwell microtiter plates precoated with anti-MMP-2 or anti-MMP-9 antibodies. Human pro-MMP-2 and pro-MMP-9, respectively, activated with p-aminophenylmercuric acetate, were used in six serial dilutions, as standard, in each plate. The detection enzyme was the proform of a modified urokinase, an enzyme that can be activated by captured active MMPs in an active detection enzyme. The natural activation sequence in the prodetection enzyme was replaced using protein engineering, with an artificial sequence recognized by specific MMP. Activated urokinases were then measured using a specific chromogenic substrate (S-2444*™*). The amount of active MMP-2 or active MMP-9 in all samples was determined by interpolation from a standard curve. According to the manufacturer's instructions, for the MMP-2 activity assay, the lower limit of quantification was 0.19 ng/mL, the range of intra-assay coefficient of variations (CV) was 4.4–7.0%, and the range of interassay CV was 16.9–18.5%, while for the MMP-9 activity assay the lower limit of quantification was assumed at 0.125 ng/mL, the range of intra-assay CV was 3.4–4.3%, and the range of interassay CV was 20.2–21.7%.

### 2.3. TIMP-1 and TIMP-2 Detection Assays

As previously described [30, 31], serum levels of TIMP-1 and TIMP-2 were measured using commercially available “sandwich” ELISA kits (Biotrak, Amersham Biosciences, Little Chalfont, UK; codes RPN2611 and RPN2618, resp.) according to the manufacturer's instructions. All the reagents and standards were included in the kits. The limit of sensitivity in both the assays was 3.13 ng/mL.

### 2.4. Data Analysis

Statistical analysis was performed with GraphPad Prism®. The normality of each variable was checked by using the Kolmogorov-Smirnov test. When normality of data distribution was found in all variables, statistical analysis was performed by a parametric approach. Accordingly, ANOVA test was used to compare variables among the various groups, and when significant differences were found, Student's *t*-test was used for the comparison between two groups. On the other hand, when normality of data distribution was rejected, statistical analysis was performed by a nonparametric approach. Kruskal-Wallis test was used to compare variables among the various groups and if significant differences were found, Mann-Whitney *U*-test was then used to compare two different groups. In case of multiple comparisons, a Bonferroni post hoc correction was applied. A value of *p* < 0.05 was accepted as significant.

## 3. Results

**Figure 1:**
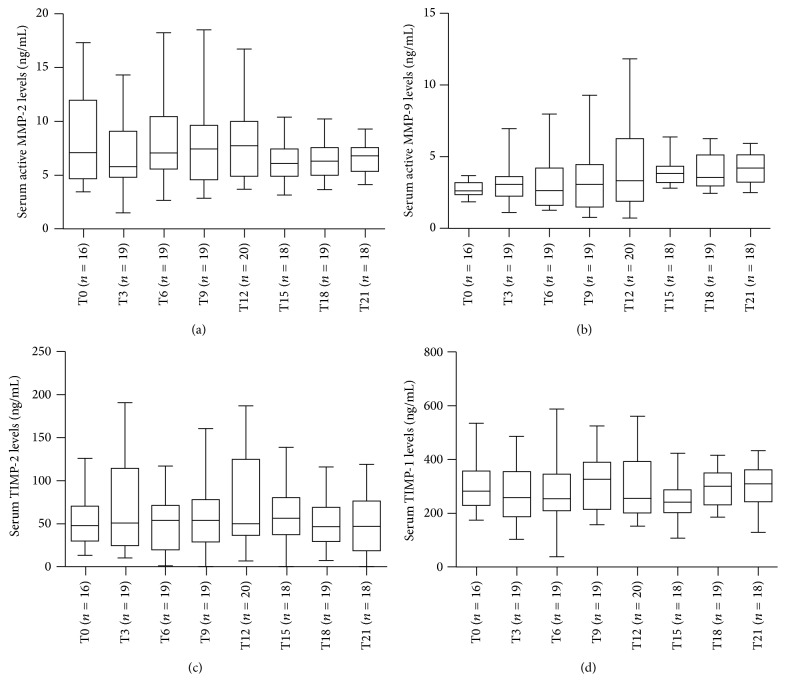
Longitudinal fluctuations of serum active MMP-2 (a) and active MMP-9 and (b) TIMP-2 (c) and TIMP-1 (d) in patients with relapsing-remitting multiple sclerosis (RRMS) treated with Natalizumab for 21 months. MMP = matrix metalloproteinases; TIMP = tissue inhibitors of metalloproteinases; T0 = baseline; T3 = 3rd month; T6 = 6th month; T9 = 9th month; T12 = 12th month; T15 = 15th month; T18 = 18th month; and T21 = 21st month. Horizontal bars indicate medians and error bars correspond to interquartile range. The boundaries of the box represent the 25th–75th quartiles. The line within the box indicates the median. The whiskers above and below the box correspond to the highest and lowest values, excluding outliers.

**Figure 2:**
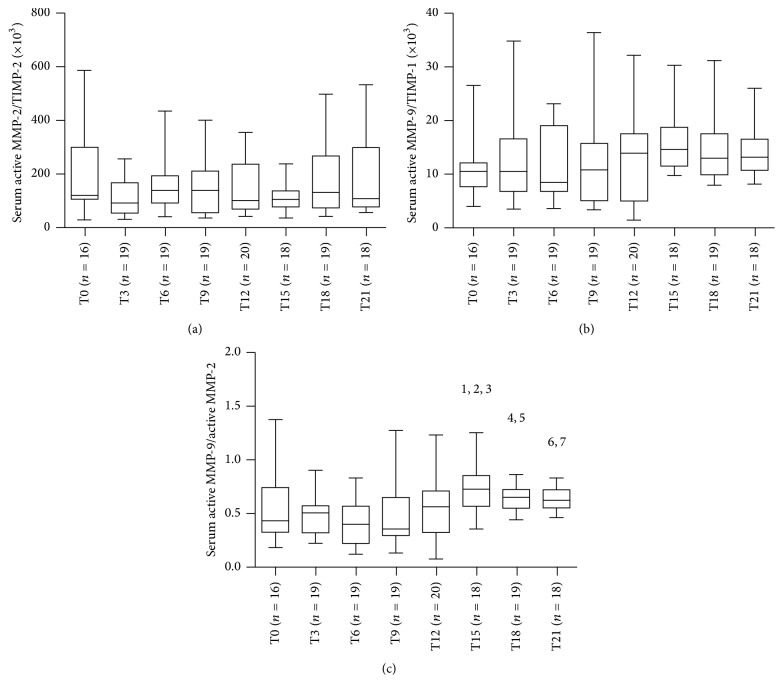
Longitudinal fluctuations of serum active MMP-2/TIMP-2 ratio (a), serum active MMP-9/TIMP-1 ratio (b), and serum active MMP-9/active MMP-2 ratio (c) in relapsing-remitting multiple sclerosis (RRMS) patients during 21 months of Natalizumab treatment. No differences were found for the MMP-2/TIMP-2 (a) and MMP-9/TIMP-1 (b) ratios while the active MMP-9/active MMP-2 ratio was different at various time points ((c), *p* < 0.001); in particular it was higher at the 15th month than at the 3rd (^1^ *p* < 0.01), 6th (^2^ *p* < 0.01), and 9th months (^3^ *p* < 0.05), increased at the 18th month than at the 3rd and 6th (^4,5^ *p* < 0.05), and more elevated at the 21st month of treatment than at the 3rd and 6th months (^6,7^ *p* < 0.05). MMP = matrix metalloproteinases; TIMP = tissue inhibitors of metalloproteinases; T0 = baseline; T3 = 3rd month; T6 = 6th month; T9 = 9th month; T12 = 12th month; T15 = 15th month; T18 = 18th month; and T21 = 21st month. Horizontal bars indicate medians and error bars correspond to interquartile range. The boundaries of the box represent the 25th–75th quartiles. The line within the box indicates the median. The whiskers above and below the box correspond to the highest and lowest values, excluding outliers.

Demographic and clinical characteristics of 20 RRMS patients treated with Natalizumab are listed in Table 1. During the therapy five patients experienced relapses (3 patients had 1 relapse between baseline and 3 months, one had 2 relapses between 6 and 9 months and at 12 months, and one had 2 relapses between 9 and 12 months and between 18 and 21 months) and four patients showed new T2 and/or Gd-enhancing lesions on the last MRI examination at the 21st month. No patients showed anti-JCV seroconversion during the 21 months of Natalizumab treatment. The timing of sample collection was not sequential and resulted incomplete for ten patients. However, we decided to analyze all the variables in RRMS patients considered as a whole. Serum levels of active MMP-2, active MMP-9, and TIMP-1 were detected in all samples, while serum levels of TIMP-2 were measured in 145/148 (98%) of samples. As reported in Figure 1, no differences were found for serum levels of active MMP-2 (panel (a), ANOVA: n.s.) and active MMP-9 (panel (b), Kruskal-Wallis: n.s.) and TIMP-2 (panel (c), Kruskal-Wallis: n.s.) and TIMP-1 (panel (d), ANOVA: n.s.) among the various time points. The ratios between MMPs and the specific tissue inhibitors and between active MMP-9 and active MMP-2 were then calculated for all the patients at each time point (Figure 2). No differences were found for the MMP-2/TIMP-2 (panel (a), Kruskal-Wallis: n.s.) and MMP-9/TIMP-1 (panel (b), Kruskal-Wallis: n.s.) ratios while the active MMP-9/active MMP-2 ratio was different at various time points (panel (c), Kruskal-Wallis: *p* < 0.001) and in particular it was higher at the 15th month (Mann-Whitney with Bonferroni correction) than at the 3rd (*p* < 0.01), 6th (*p* < 0.01), and 9th months (*p* < 0.05), more elevated at the 18th month than at the 3rd and 6th (*p* < 0.05), and finally more increased at the 21st month of treatment than at the 3rd and 6th months (*p* < 0.05). Afterwards, we tried to compare patients who were free of relapses during the treatment, considered as “responders,” with patients who experienced at least one relapse, “nonresponders.” Despite the small number of patients in each group, we compared all the variables: serum concentrations of active MMP-2 and active MMP-9 and TIMP-2 and TIMP-1 and the ratios calculated between MMPs and TIMPs and between active MMP-9 and active MMP-2. No differences were found between the responders and the nonresponders for all the data analyzed (data not shown).

## 4. Discussion

To the best of our knowledge, this is the first study that longitudinally analyzes serum levels of active MMP-2 and active MMP-9 with sensitive activity assay systems in a cohort of RRMS patients during the treatment with Natalizumab in an attempt to provide further insight into the real significance of gelatinases in MS pathology and their role in monitoring efficacy of treatment. The involvement of MMP-2 and MMP-9 in MS pathogenesis and progression has been widely investigated in the past decades. There is a large agreement, particularly on the proinflammatory role of MMP-9 and on the protective function of TIMP-1. In particular, serum MMP-9 levels were greater in RRMS than in the progressive forms [14–16] in MS patients with MRI evidence of disease activity [15, 17, 19] and in MS subjects with clinically isolated syndromes who developed clinically definite MS [18]. On the other hand, TIMP-1 levels were lower in MS patients than in controls [14, 15, 17] and serum MMP-9/TIMP-1 ratio has been indicated as a potential biomarker of MS disease activity [15, 18]. The study of the active forms of MMP-9 also demonstrated that CSF and serum levels of active MMP-9 could represent a potential biomarker for monitoring MS disease activity and that serum active MMP-9/TIMP-1 ratio seems to be an indicator of ongoing MS inflammation [30]. In addition, beneficial effects of Natalizumab treatment were associated with decreased CSF MMP-9 levels after 12 months of therapy, and for this reason MMP-9 was proposed as a biomarker for clinical trials on new drugs for MS [21]. On the contrary, the role of MMP-2 still remains unclear. Previous studies have reported that MMP-2 was elevated in PBMCs and CD4+ Th1 cells of MS patients and could contribute to the homing of these immune cells inside the brain through the BBB [28, 29]. In addition, while CSF MMP-2 levels appeared to be comparable between MS and controls [14, 15, 22, 25, 26], serum MMP-2 concentrations were similar or lower in MS patients than in controls [15, 20]. The active form of MMP-2 has been described as a potential marker of MS recovery, as detected by MRI, suggesting a beneficial function that sustains the resolution of the inflammatory response and the remission of the disease [31]. In the present study, serum levels of active MMP-2 and active MMP-9 and of the specific tissue inhibitors TIMP-2 and TIMP-1, respectively, were found to be stable during the 21 months of Natalizumab therapy in all patients. Surprisingly, despite the small number of patients included in the study, we did not find differences between patients who experienced relapses during the treatment, considered as “nonresponders,” and patients that were free of relapses, considered as “responders,” for active MMP-2 and active MMP-9 and TIMP-2 and TIMP-1 serum levels at each time point. Moreover, the ratios between active MMPs and the respective TIMPs did not appear influenced by the Natalizumab treatment and did not differ between responders and nonresponders during the 21 months of observation. On the one hand, this could indicate that Natalizumab treatments maintain stable serum levels of MMPs and TIMPs, but, on the other hand, this excludes the use of these molecules in monitoring the pharmacological response. Previous studies on recombinant interferon beta-1a, one of the most used disease-modifying therapies for MS, showed that low MMP-9 serum levels were associated with a positive outcome, while MMP-2 serum levels were stable during treatment [36] and that serum MMP-9/TIMP-1 ratio may be regarded as a reliable marker and may be predictive of MRI activity in RRMS [37]. Moreover, TIMP-1 has also been suggested as a good indicator of response to therapy [38]. Our principal finding was that an imbalance occurred between active MMP-9 and active MMP-2 serum levels after 15 months of Natalizumab therapy without further differences between responder and nonresponder patients. The ratio between MMP-9 and MMP-2 was proposed as a serum marker to monitor the progression of liver disease, and the ratio between MMP-2 and MMP-9 was associated with poor response to chemotherapy in osteosarcoma in two previous studies [39, 40]; however this is the first time that the ratio between the active forms of MMP-9 and MMP-2 was calculated in MS patients, and for this reason further studies are required to investigate the biological significance of the imbalance between serum levels of gelatinases. The main limitations of this study were the small number of enrolled patients and above all the lack of samples collected at the time of relapse. In conclusion, taken together, our data seems to indicate that Natalizumab treatment could either maintain serum levels of active MMP-2 and active MMP-9, as well as of the specific tissue inhibitors TIMP-1 and TIMP-2, stable or make them not affected at all; however, this influence tends to be reduced after 15 months of therapy resulting in an imbalance between serum active MMP-9, considered as a marker of disease activity [30], and serum active MMP-2, described as a marker of disease remission [31]. Moreover, the present study argues against the use of serum levels of gelatinases for the monitoring of Natalizumab treatments in MS patients. Nevertheless, more extensive research in a larger number of patients is advisable for a better understanding of the correlations between Natalizumab therapy and gelatinases in MS.

## Figures and Tables

**Table 1:** Demographic and clinical characteristics of 20 RRMS patients stratified according to response to therapy before and during treatment with Natalizumab.

	Responders	Nonresponders
Patients (*n*)	15	5
Sex (male/female)	3/12	0/5
Age at entry, years (mean ± SD)	35.1 ± 10.1	31.6 ± 9.4
EDSS at baseline (mean ± SD)	1.0 ± 1.1	2.3 ± 2.4
EDSS after 21 months of therapy	1.3 ± 1.3	2.8 ± 2.4
Relapses during 21 months of therapy (mean ± SD)	0	1.6 ± 0.9
Patients with new MRI lesions at the end of treatment	4	0

EDSS = Expanded Disability Status Scale; MRI = magnetic resonance imaging; SD = standard deviation.
